# Rhizosphere microbiome assembly drives metal sequestration in *Leucaena leucocephala* during tailing phytoremediation

**DOI:** 10.3389/fmicb.2026.1745018

**Published:** 2026-02-13

**Authors:** T. Emmanuel Doku, J. D. Ebenezer Belford, A. Augustina Sylverken

**Affiliations:** 1 Department of Pharmaceutical Sciences, Sunyani Technical University, Sunyani, Ghana; 2 Department of Theoretical and Applied Biology, Kwame Nkrumah University of Science and Technology, Kumasi, Ghana

**Keywords:** *Leucaena leucocephala* (lam.) de wit, metal sequestration, mine tailing, phytoremediation, rhizosphere microbiome

## Abstract

**Introduction:**

Ghana’s water and soil resources face severe challenges due to heavy metal contamination from gold mining operations. Although *Leucaena leucocephala* exhibits potential for phytoremediation, little is known about the contribution of its rhizosphere microbiomes to metal uptake and tolerance in multiple-metal contaminated tailings in field conditions.

**Methods:**

We investigated the rhizosphere bacterial community dynamics in *L. leucocephala* across three soil treatments (garden soil, 1:1 soil-tailings mixture, and pure tailings) using 16S rRNA amplicon sequencing and atomic absorption spectrophotometry. Briefly, transplanted seedlings of *L. leucocephala* were harvested at three-month intervals for three consecutive harvests to assess metal accumulation and changes in the microbiome.

**Results and discussion:**

*Leucaena leucocephala* demonstrated notable tolerance to elevated metal concentrations (>10,000 mg/kg Fe and Mn) under acidic conditions (pH 4.57–5.97). Maximum metal uptake occurred at final harvest, with Fe reaching 14,605 ± 1.40 mg/kg in shoots and Mn reaching 12,279 ± 1.13 mg/kg in roots. The elevated concentrations of metals reduced overall bacterial diversity, except for selected metal-tolerant *Actinobacteria*, *Proteobacteria*, and *Acidobacteria*, which dominated bacterial communities across all treatments. The initial proliferation of *Nocardioides* and *Streptomyces* corroborated nutrient and metal-induced stress, while key genera such as *Arthrobacter*, *Gaiella*, *Skermanella*, *and Chelatococcus* showed strong positive associations with metal accumulation and maintained essential ecological functions.

**Conclusion:**

Rhizosphere bacterial communities undergo stress-specific assembly processes, with specific taxa facilitating *L. leucocephala’s* exceptional phytoremediation capacity. These findings provide insights into microbiome-enhanced strategies for mine site rehabilitation.

## 1 Introduction

Gold mining activities in Ghana have intensified over the past decade, making a significant contribution to the country’s economic development (Aram et al., 2021; Duncan, 2020; Yiridomoh, 2021). However, these mining activities generate tonnes of wastes called tailings, which contain elevated high concentrations of heavy metal ions such as copper (Cu), iron (Fe), arsenic (As), cadmium (Cd), manganese (Mn), and zinc (Zn; Bharti and Sharma, 2021; Hadzi et al., 2019; Islam and Murakami, 2021; Kossoff et al., 2014). Heavy metals pose serious threats to ecosystem health, soil fertility, water quality and long-term health of individuals living in surrounding communities (Duncan, 2020; Idemudia et al., 2020; Obuobi et al., 2022; Kumi et al., 2024).

The utilisation of plants to stabilise, sequestrate, or transform contaminants in the environment, referred to as phytoremediation, has gained much attention compared to other conventional methods due to its ecologically friendly and sustainable nature (Bomfim et al., 2021; Jia et al., 2022; Kidd et al., 2009; Sabreena et al., 2022). The effectiveness of the phytoremediation of heavy metals in mine tailings depends on multiple interconnected factors, including the concentration of heavy metals, the extent of plant metal tolerance, the physicochemical properties of the tailing, levels of nutrients, and the presence of microbial communities capable of transforming heavy metals (Alves et al., 2022; Di Carlo et al., 2019; Huang et al., 2012; Korkar et al., 2022).

*Leucaena leucocephala* (Lam.) de Wit, a fast-growing leguminous shrub, has emerged as a promising candidate for the phytoremediation of tailings due to its exceptional tolerance to elevated metals concentrations, aggressive growth, high biomass production, deep and extensive rooting system and nitrogen-fixing capacity that can improve soil fertility (Couic et al., 2022; Garcia et al., 2020; Saraswat and Rai, 2011). Previous studies have demonstrated its capacity for the phytostabilisation of multiple metal-contaminated tailings; however, most of these investigations have focused primarily on physiological responses and metal stabilisation patterns without providing insights into the rhizospheric microbial community dynamics underlying this remarkable performance (Nkongolo et al., 2025; Asif et al., 2025; Doku et al., 2024; Garcia et al., 2020; Petelka et al., 2019).

The narrow zone between the root surface and the surrounding soil, the rhizosphere, harbours diverse and metabolically active microbial communities that can influence phytoremediation outcomes via a plethora of mechanisms (biotransformation, nutrient cycling, hormone production and growth promotion; Huang et al., 2021; Mishra et al., 2017; Qu et al., 2022). However, most studies have focused on single or a few contaminant profiles, leaving a significant knowledge gap regarding bacterial community responses to multiple metal-contaminated tailings in field conditions (Duan et al., 2021; Gao et al., 2021; Guo et al., 2019; Saldarriaga et al., 2023; Sun et al., 2024).

This study addresses a vital knowledge gap by investigating the response of the rhizosphere bacterial communities of *L. leucocephala*, which facilitates the phytoremediation of multiple metal-contaminated mine tailings over time. Thus, the specific objectives are to: (1) characterise changes in bacterial community structure and diversity across metal contamination gradients, (2) identify key bacterial taxa associated with enhanced metal uptake and plant performance, (3) elucidate ecological network relationships that maintain ecosystem function under metal stress, and (4) provide insights for the developing microbiome-enhanced phytoremediation strategies. This research represents an initial comprehensive temporal analysis of plant-microbe interaction in the *Leucaena leucocephala* phytoremediation system.

## 2 Materials and methods

### 2.1 Study site and soil sample collection

Heavy metal-contaminated tailings were sourced from the AngloGold Ashanti mine tailings dam at Pompora, Obuasi (1°3,925”–1°38’24” W and 6°13′11”–6°13’19” N), Ghana. The tailings are old residues from gold extraction activities that used cyanide leaching and flotation methods. Uncontaminated soil was sourced from the botanical garden of the Kwame Nkrumah University of Science and Technology, Kumasi, representing typical tropical agricultural soil with no history of mining.

### 2.2 Experimental design and setup

The experimental design comprised three treatments: garden soil only (GS), 1:1 [mixture of garden soil and tailings (w/v), and pure tailings (PT)]. All soil samples were sieved through a 1 mm sand filter before being weighed into respective pots, each weighing 5 kg. Before the field experiment, the soils were watered to field capacity and initially kept in a greenhouse with natural light, with daytime temperatures ranging from 27 °C to 32 °C.

Four harvest timepoints were established: initial setup after transplanting (H0) and three successive harvests at three-month intervals (H1, H2, H3). Triplicate pots were set up randomly for each treatment (GS, 1:1, and PT) per harvest and arranged in a completely randomised design, totalling 36 experimental samples.

### 2.3 Plant material and growth conditions

Seeds of *L. leucocephala* were collected from the Pompora tailings site and stored in a dry container. Seeds were initially germinated in nursery pots containing only garden soil watered to field capacity and kept in a greenhouse. Seeds germinated at an average of 3 days and thinned to one seedling per pot. The seedlings were grown for 2 weeks after germination and then introduced into the experimental treatment pots, which were kept under natural light and temperature conditions in an open field.

### 2.4 Rhizosphere sampling

Rhizosphere samples were obtained by uprooting plants from treatment pots, gently shaking them to remove bulk soil, and placing them into sterile bags. Rhizosphere samples were collected by vigorously shaking the tightly bound soil attached to the roots, placed in sterile sampling bags. Triplicates of each sample were kept separately for physicochemical and heavy metal analyses. For molecular analysis, triplicates were pooled per treatment to obtain a composite sample representing each treatment-harvest combination, which was stored at −80 °C for 1 month before DNA extraction.

### 2.5 Plant tissue collection

Plant samples were thoroughly washed under slow-running water to remove particles and air-dried for 24 h. Both soil and plant samples designated for heavy metal analysis were oven-dried at 50 °C to a constant weight.

### 2.6 Physicochemical and heavy metal analyses

#### 2.6.1 Soil parameters analysis

Fresh rhizosphere samples (5 g) were suspended in 100 mL of deionised water to determine pH and electrical conductivity using a calibrated HACH Sension-plus multiple-parameter probe. The nutrient analysis included nitrate-nitrogen (Kjeldahl method), total phosphate (barium chloride titration), and available phosphorus (Bray-1 method). Cation exchange capacity was determined using the cation displacement method, while the percentage organic matter was determined by the Walkley-Black method.

#### 2.6.2 Heavy metal analysis

Dried soil and plant samples were milled and subjected to acid digestion (HNO_3_ and HClO_4_; 3:1 v/v) and analysed for Fe, Mn, As, Cd, Cu, and Zn concentrations using atomic absorption spectrophotometry (VGP 210 FAAS, Buck Scientific, United States). Quality control included certified reference materials and analytical blanks for each of the 10 samples. The limits of detection are as follows (mg/L): Fe (0.005–0.01), Mn (0.001–0.002), As (0.1–0.05), Cd (0.001–0.002), Cu (0.002–0.005) and Zn (0.001–0.005).

#### 2.6.3 DNA extraction and sequencing

Total DNA was extracted from a 0.5 g rhizosphere sample using the PowerSoil DNA isolation kit (QIAGEN) following the manufacturer’s instructions. DNA quality and concentration were assessed using NanoDrop spectrophotometry and gel electrophoresis. The hypervariable region (V3-V4) of the 16S rRNA was amplified using universal primers 341F and 805R. PCR products were purified, quantified and pooled for paired-end sequencing (2 × 300 bp) on the Illumina MiSeq platform, Beijing Genomics Institute, Hong Kong.

#### 2.6.4 Data processing, community structure and network analyses

A total of 819,271 raw sequence reads were processed using DADA2 implemented in QIIME2 version 2023.2 (Callahan et al., 2016). Quality trimming based on the following parameters (forward—260 bases and reverse—230 bases) yielded 442,086 high-quality reads, which were classified using the Greengenes2 database on a trained naïve Bayesian classifier (McDonald et al., 2022). The reads were subsampled to a depth of 26,330, and low-abundance reads (< 0.1%) were removed during pre-processing in the Microeco R package (Liu et al., 2021). Functional prediction of bacterial communities was conducted in QIIME2-picrust2, and further analysis was performed in R using ggpicrust2 (Yang et al., 2023). Alpha diversity measures were calculated using observed ASVs, ACE, Chao1, Shannon, and Simpson indices. Beta diversity was assessed using Bray–Curtis dissimilarity with PERMDISP and PERMANOVA tests for significant differences among treatments and Harvests. An ecological network was constructed using random matrix theory (RMT) with default parameters (Deng et al., 2012). Network topology was analysed to identify keystone species and modular structure, and visualised in Cytoscape v3.10.3.

### 2.7 Statistical analysis

The results of the physicochemical parameters and heavy metal concentrations were analysed using SPSS version 22. The statistical differences in the mean values of the measures were distinguished using the Tukey HSD test at a 95% confidence interval. Correlations between bacterial communities and environmental variables were analysed using canonical correspondence analysis (CCA) in PAST 4.0.

### 2.8 Bioaccumulation of heavy metals

The bioaccumulation factor (BAF) of heavy metals in plant tissues was estimated as follows: BAF = Xt/ Xs, where Xt and Xs represent the concentration of heavy metals in plant tissues and soil, respectively.

### 2.9 Translocation of heavy metals

The translocation factor (TF) of heavy metals in plant tissues was estimated as follows: TF = X_shoot / X_root, where X_shoot and X_root represent the concentration of heavy metals in shoot and root tissues, respectively.

## 3 Results

### 3.1 Physicochemical changes during phytoremediation

Rhizospheric physicochemical properties of the rhizosphere changed significantly during phytoremediation (*p* < 0.05; Table 1). The pH remained acidic across all treatments, with values ranging from 4.57 ± 0.10 to 0.97 ± 0.03, and reduced gradually over time (H0–H4). Specifically, pure tailings showed the most acidic conditions, while garden soil maintained relatively higher pH values. Electrical conductivity was highest at the onset (H0) across all treatments and decreased progressively over time, ranging between 102.50 ± 3.53 μS/cm and 291.00 ± 0.14 μS/cm. Nutrient levels varied significantly among treatments and over time (*p* < 0.05; Table 1). Available phosphorus ranged from 28.73 ± 1.03 mg/kg to 65.89 ± 1.25 mg/kg, with garden soil consistently showing higher levels than tailing treatments. Total nitrogen content was lowest in pure tailings at the onset and first harvest (0.12 ± 0.03 mg/kg, H0 and H1) and highest in garden soil treatments (0.27 ± 0.002–0.35 ± 0.05). Interestingly, sulphate levels were elevated in tailing treatments (37 ± 0.00 mg/kg–47 ± 1.40 mg/kg) compared to garden soil (14.50 ± 2.12 mg/kg–21.50 ± 2.12 mg/kg), reflecting the sulphur-rich mineralogy of the mine tailings. The cation exchange capacity of the rhizosphere was relatively high in garden soil, with values increasing to the second harvest, which recorded the highest value (26.32 ± 1.47 cmol/Kg), followed by a decline to 16.24 ± 0.34 cmol/Kg. No significant changes in cation exchange capacity were observed between H0–H2 for the 1:1 treatment, with a significant increase at H3 (16.20 ± 0.33). The percentage of organic matter in the rhizosphere reduced at first harvest but increased gradually subsequently across different treatments, with values ranging 1.94 ± 0.03–2.24 ± 0.07, in garden soil, followed by 1:1 (1.24 ± 0.02–1.82 ± 0.04) and pure tailing (0.41 ± 0.02–1.03 ± 0.02) in descending order.

**Table 1:** Physicochemical parameters in the rhizosphere during phytoremediation.

Treatment	Harvest	Conductivity (μS/cm)	pH	Available phosphorus (mg/Kg)	Total nitrogen (mg/Kg)	Total sulphate (mg/Kg)	CEC (cmol/Kg)	Organic matter (%)
Garden soil	H0	130.50 ± 0.71d	5.97 ± 0.03c	65.89 ± 1.25f	0.33 ± 0.05c	20.50 ± 0.71b	15.49 ± 0.35 cd	2.23 ± 0.06 k
	H1	149.00 ± 1.41f	5.33 ± 0.04b	36.92 ± 1.30e	0.35 ± 0.04c	21.50 ± 2.12b	17.02 ± 0.37e	1.93 ± 0.03i
	H2	121.00 ± 1.40c	5.28 ± 0.40b	45.89 ± 1.25d	0.27 ± 0.02bc	20.00 ± 1.41b	26.32 ± 1.47f	2.13 ± 0.05j
	H3	111.00 ± 1.44b	5.38 ± 0.20b	36.18 ± 1.67bc	0.29 ± 0.03bc	14.50 ± 2.12a	16.24 ± 0.34d	2.24 ± 0.07 k
1:1	H0	121.00 ± 1.35c	4.60 ± 0.14a	40.88 ± 1.24e	0.22 ± 0.03b	33.00 ± 1.40d	14.19 ± 0.30c	1.82 ± 0.04 h
	H1	181.00 ± 1.50 h	4.68 ± 0.25a	35.57 ± 0.81bc	0.21 ± 0.01b	32.50 ± 0.71d	14.35 ± 0.31c	1.24 ± 0.02e
	H2	102.50 ± 3.53a	4.71 ± 0.16a	38.82 ± 1.16 cd	0.26 ± 0.01bc	30.50 ± 0.75 cd	14.11 ± 0.31c	1.65 ± 0.03 g
	H3	111.00 ± 1.40b	4.66 ± 0.08a	34.94 ± 1.32bc	0.34 ± 0.01c	27.50 ± 0.10c	16.20 ± 0.33d	1.55 ± 0.04f
Pure tailings	H0	171.00 ± 0.35 g	4.57 ± 0.10a	36.19 ± 1.68bc	0.12 ± 0.02a	47.00 ± 1.40f	14.96 ± 0.24c	1.03 ± 0.02d
	H1	291.00 ± 0.140i	4.74 ± 0.06a	34.81 ± 0.27bc	0.12 ± 0.03a	44.00 ± 1.45f	11.46 ± 0.26b	0.41 ± 0.02a
	H2	151.00 ± 1.50f	4.68 ± 0.11a	32.84 ± 1.19b	0.14 ± 0.02a	39.00 ± 1.50e	10.79 ± 0.20a	0.48 ± 0.01b
	H3	141.00 ± 0.50e	4.58 ± 0.15a	28.73 ± 1.03a	0.22 ± 0.03b	37.00 ± 0.40e	11.92 ± 0.22b	0.55 ± 0.01c

Mean values (means ± standard error) with different letters indicate significant differences (*p* < 0.05, Tukey’s HSD test).

### 3.2 Heavy metal levels in rhizosphere

The concentration of iron (Fe) was the highest among all metals studied, ranging from 126.00 ± 1.40 to 205,142.00 ± 2.82 mg/kg (Table 2). Metal concentrations in the rhizosphere followed a contamination gradient consistently (PT > 1:1 > GS) and decreased progressively from the onset to the final harvest (H0 > H1 > H2 > H3) across all treatments. Manganese levels were the second highest concentrations (32.50 ± 0.72–10,014.50 ± 1.70 mg/kg), while the levels of Cu, Zn, As, and Cd ranged between (31.70 ± 0.42–788.30 ± 2.44 mg/kg), Zn levels (5.00 ± 0.28–298.00 ± 1.41 mg/kg), As levels (0.30 ± 0.05–110.61 ± 0.56), and Cd levels (0.39 ± 0.01–2.45 ± 0.01 mg/kg), respectively.

**Table 2:** Mean concentrations of heavy metals in the rhizosphere of *L. leucocephala* during phytoremediation.

Treatment	Harvest	Fe	Zn	Cd	Cu	As	Mn
Garden soil	H0	352.50 ± 3.54d	13.5 ± 0.71d	0.07 ± 0.04d	87.90 ± 1.56d	0.48 ± 0.01c	79.00 ± 0.14d
	H1	321.00 ± 1.40c	9.75 ± 0.35c	0.04 ± 0.01a	57.85 ± 0.50c	0.37 ± 0.04ab	59.00 ± 0.10c
	H2	271.00 ± 1.42b	5.00 ± 0.28b	0.05 ± 0.01bc	49.35 ± 1.01b	0.34 ± 0.09ab	35.50 ± 0.70b
	H3	126.00 ± 1.40a	3.55 ± 0.64a	0.06 ± 0.04bc	31.70 ± 0.42a	0.30 ± 0.05a	32.50 ± 0.72a
1:1	H0	119,952.50 ± 3.54i	298.00 ± 1.41 k	2.15 ± 0.01j	788.30 ± 0.44 L	54.87 ± 0.52 g	8,303.50 ± 2.12 k
	H1	104,151.00 ± 1.40 h	116.50 ± 0.71i	1.63 ± 0.10i	560.50 ± 0.71j	52.17 ± 0.10f	7,177.00 ± 1.40 h
	H2	91,260.50 ± 0.71 g	50.95 ± 1.34 g	0.98 ± 0.02f	276.90 ± 0.14f	48.92 ± 1.30e	6,824.50 ± 0.70 g
	H3	15,540.50 ± 0.70e	50.60 ± 0.85 g	0.49 ± 0.01e	254.35 ± 0.50e	32.27 ± 0.38d	3,149.00 ± 1.40e
Pure tailings	H0	205,142.00 ± 2.82 L	282.50 ± 0.71j	2.45 ± 0.01 k	625.75 ± 0.35 k	110.61 ± 0.56 k	10,014.50 ± 1.70 L
	H1	197,641.00 ± 1.40 k	102.50 ± 0.70 h	1.67 ± 0.01i	462.80 ± 0.28i	98.77 ± 0.33j	8,260.50 ± 0.70j
	H2	181,401.00 ± 1.42j	40.80 ± 0.28f	1.37 ± 0.01 h	456.60 ± 0.57 h	94.71 ± 0.42i	7,615.50 ± 0.72i
	H3	30,751.00 ± 1.45f	36.85 ± 0.21e	1.31 ± 0.02 g	394.45 ± 0.78 g	89.76 ± 0.35 h	4,879.00 ± 1.40f

Mean values (means ± standard error) with different letters indicate significant differences (*p* < 0.05, Tukey’s HSD test).

### 3.3 Metal accumulation in plant tissues

*Leucaena leucocephala* (Lam.) de Wit demonstrated notable capacity for metal tolerance and uptake across all treatments. Distinguished accumulation of Fe was observed in shoot tissues (14,605 ± 1.40 mg/kg) in pure tailings at the final harvest (H3), which is above the hyperaccumulation threshold of 10,000 mg/kg (Supplementary Table 2). Conversely, manganese showed preferential accumulation in roots, with maximum concentrations of 12,279.20 ± 1.13 mg/kg occurring in the 1:1 treatment (Supplementary Table 1). Similarly, superior accumulation in root tissues was observed for arsenic and cadmium, indicating limited translocation to aboveground or shoot tissues. Alternatively, the accumulation of Zn was higher in shoot tissues (24.35 ± 0.21–4,699.10 ± 1.40 mg/kg) along an increasing contamination gradient (GS < 1:1 < PT), which is typical of micronutrients.

### 3.4 Bacterial community structure and diversity

#### 3.4.1 Alpha diversity

Bacterial community diversity showed complex temporal patterns influenced by metal contamination levels (Supplementary Table 3). Species richness (ACE, Chao1, Observed ASVs) gradually increased from the first harvest (H1) to the third harvest (H3) in 1:1 treatments. In contrast, peak diversity occurred at H1, followed by a temporally based decline in pure tailing. Shannon diversity ranged from 6.91 to 5.63, with pure tailing consistently maintaining higher diversity across H0–H2.

#### 3.4.2 Beta diversity and community composition

Beta diversity of bacterial communities in the rhizosphere of *L. leucocephala* revealed distinct microbiomes along a contamination gradient (Bray-Curtis PERMANOVA: *F* = 8.42, *p* < 0.001, R^2^ = 0.65). Pure tailings communities were distinguished from garden soil and mixed treatments, demonstrating significant shifts in bacterial community assembly along the contamination gradient (PT > 1:1 > GS; Supplementary Figure 1).

**Figure 1:**
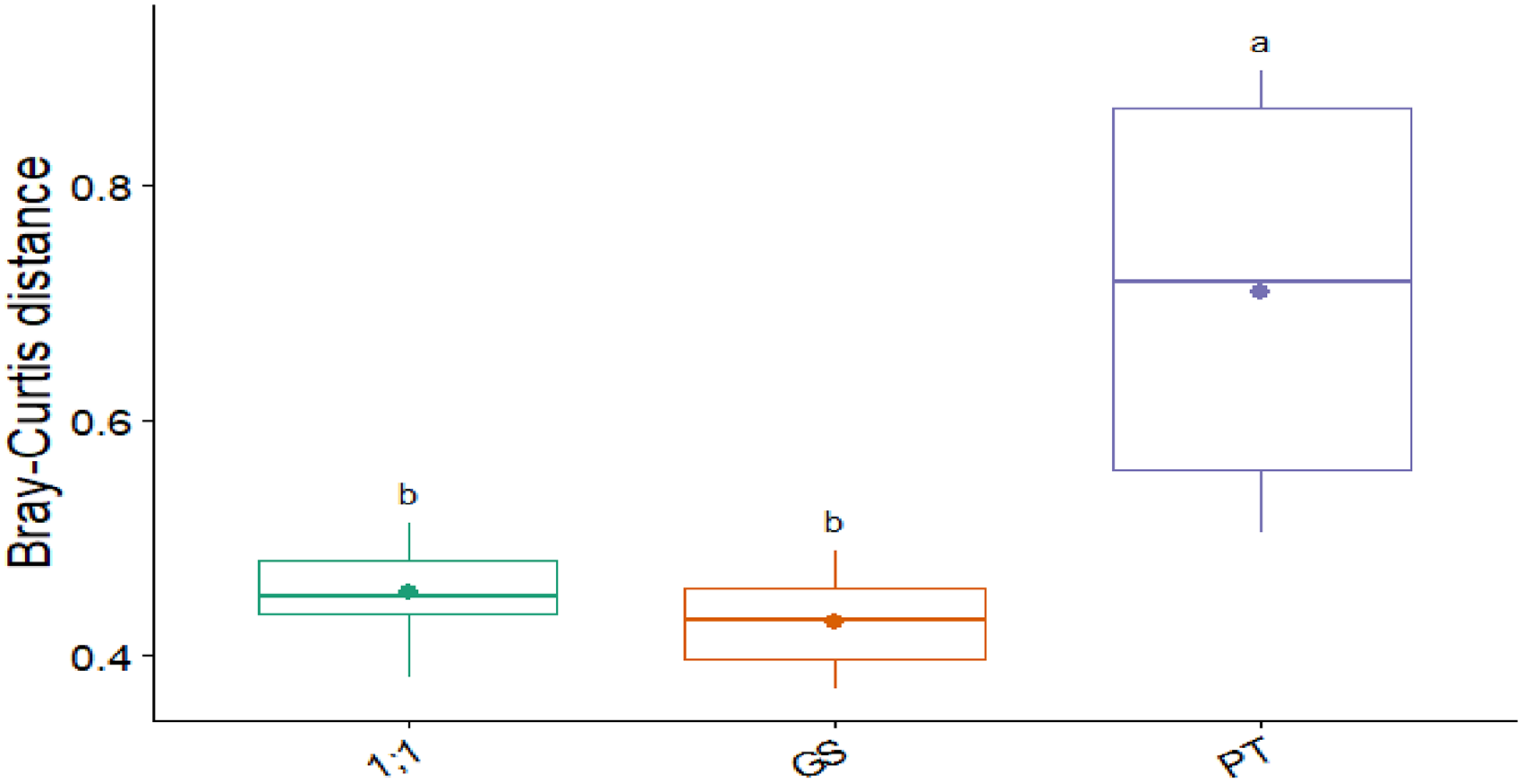
Relative abundance of bacterial phyla during phytoremediation.

*Actinobacteria*, *Proteobacteria*, and *Acidobacteria* constituted predominant bacterial phyla (70% of total abundance) across all treatments (Figure 1). Specifically, *Actinobacteria* maintained relatively stable community proportions across all treatments and harvest periods (32.8–37.3%). Additionally, the minor phyla showed more variable patterns relative to the extent of contamination and harvest period (Supplementary Figures 2a,b).

Heatmap of the relative abundance of bacterial communities elucidated the distinct responses of *Gaiella*, *Arthrobacter*, *Nocardioides*, *Streptomyces*, *Mycobacterium*, *Solirubacter*, *Desulfomonas*, and *Ilumatobacter* to contamination gradients and temporal changes during phytoremediation (Supplementary Figure 3).

Predicted functional states of bacterial communities in the rhizosphere unveiled 489 pathways, of which branched-chain amino acid production, aerobic respiration, pyruvate fermentation, and fatty acid oxidation were predominant, especially in pure tailing treatment (Supplementary Figure 4). Predominant predicted plant-growth-promoting (PGP) functions of bacterial communities include phosphate solubilisation, vitamin production, VOC production, auxin biosynthesis, biofilm and colonisation, osmotic stress tolerance, nitrogen fixation and siderophore production, especially in pure tailing treatment (Supplementary Figure 5). A correlation heatmap of predicted PGR functions indicated a strong, significant association between Cd and most predicted functions (*p* < 0.05), and a moderate correlation of Fe with siderophore production (*p* < 0.05) in shoot accumulation (Supplementary Figure 6). On the other hand, a strong, significant association was observed between Cd, As and Zn with most predicted PGP functions in root accumulation (Supplementary Figure 7).

#### 3.4.3 Sequestration-associated bacterial taxa

Canonical correspondence analysis reveals strong associations between specific bacterial genera and patterns of metal accumulation. *Arthrobacter* and *Gaiella* showed positive correlations with Fe, Cd, As and Mn accumulation in root tissues (Supplementary Figure 8). Similarly, the accumulation of Cd, Mn and Zn in the shoot was associated with the abundance of *Mycobacterium*, *Arthrobacter* and *Gaiella* (Supplementary Figure 9).

### 3.5 Bacterial diversity patterns and environmental factors

Correlation Heatmap analysis unveiled positive associations between physicochemical parameters (pH, phosphorus, nitrogen, CEC, organic matter and sulphate levels) and bacterial diversity (Supplementary Figure 10). Additionally, metal levels in the rhizosphere correlated negatively with species richness or abundance (ACE, Chao1, Observed ASVs).

### 3.6 Ecological network analysis

Network analysis revealed three major modules comprising 81 nodes and 678 edges (Supplementary Tables 4, 5). The network showed high connectivity and moderate transitivity, indicating a well-connected community with substantial redundancy. Three bacterial genera emerged as keystone species based on network centrality metrics: *Arthrobacter* (highest degree and eigenvector centrality), *Skermanella* (high betweenness centrality) and *Chelatococcus* (high connectivity within modules). *Arthrobacter* showed the highest degree (40) and eigenvector centrality (0.235), confirming its essential role in community interactions. Despite the low abundance of *Skermanella*, it exhibited the maximum betweenness centrality (210.45), indicating its vital role as a connector between different community modules. *Actinobacteria*, *Proteobacteria* and *Acidobacteria* preferentially sustained more functions within networks as connectors and module hubs compared to minor taxa, which served as peripheral nodes within and between ecological networks (Supplementary Figure 11).

### 3.7 Bioaccumulation of heavy metals

Strong bioaccumulation of Fe and Zn occurred in roots, whereas As and Cd showed strong bioaccumulation in the shoot. Bioaccumulation of heavy metals in roots was relatively high in pure tailings and later harvests (H2–H3; Supplementary Table 6a). Zinc bioaccumulation was superior to other metals, with values ranging from 2.5–757.43, and this was followed by Mn (0.12–140.27), Fe (1.01–64.62), As (0.17–10.85), Cd (0.03–3.37), and Cu (0.51–11.51) in descending order. The bioaccumulation of metals in the shoot was relatively high in 1:1 and later harvests (H2–H3; Supplementary Table 6b). The bioaccumulation of Zn was superior to other metals, with values ranging 0.38–300.49, followed by Mn (0.77–208.12), Fe (0.09–48.82), As (0.11–34.21), Cu (0.34–12.02) and Cd (0.02–7.42) in descending order.

### 3.8 Translocation of heavy metals

The translocation of heavy metals in L. leucocephala was higher in garden soil treatments than 1:1 and pure tailings (Supplementary Table 7). In metallifoerus treatments (1:1 and pure tailings), Zn and Fe exhibited higher translocation, whereas moderate translocation was observed for As, Mn and Cu.

## 4 Discussion

### 4.1 Rhizosphere adaptation to metal stress

Abiotic factors (pH, conductivity, nutrients, and heavy metals) affect the soil’s bacterial community composition and structure (Huang et al., 2021; Liu et al., 2023; Sun et al., 2018). Across all treatments, pH remained consistently acidic (4.57–5.97), with the most extreme acidification in pure tailings. The observed acidification of the rhizosphere represents a key adaptive mechanism that enhances metal solubility and bioavailability for uptake into plant tissues (Park et al., 2023; Wan et al., 2023; Yang et al., 2022). The electrical conductivity (EC) within the rhizosphere increased at the first harvest but decreased gradually across all treatments, and coupled with acidification, suggests active modification of the rhizosphere chemical environment through root exudates, microbial metabolites and organic acid production (Amin et al., 2012; Mulati et al., 2023; Ni et al., 2019).

Progressive nutrient depletion, especially nitrogen and phosphorus, likely reflects intensive utilisation for metal transport proteins, stress response mechanisms, and cellular repair processes, which has been recognised in other research outcomes (Li Q, et al., 2022; Bertrand et al., 2021; Skuza et al., 2022; Qian et al., 2023; Asare et al., 2023). Although gold mine tailings are associated with low organic matter and nutrient levels, they contain high levels of sulphur in the mineralogical sources (pyrite, galena and chalcopyrite), providing opportunities for sulphur metabolising bacteria and challenges for other bacterial communities (Huang et al., 2012; Lottermoser, 2011; Mensah et al., 2020). Organic matter levels and CEC were higher in garden soil and 1:1 treatments than in pure tailings, although they were within normal (low–moderate) ranges characteristic of Ghanaian soils (Attiogbé et al., 2026; Doe et al., 2022). Moreover, these levels showed low–moderate associations with bacterial community diversity, highlighting the challenges faced by rhizosphere microbial communities, especially in pure tailings, which have limited carbon sources and poor nutrient retention capacity (Hu et al., 2021).

### 4.2 Plant performance and metal accumulation

*Leuceana leucocephala* demonstrated remarkable potential in the phytoextraction of metal ions in mine tailing, achieving hyperaccumulation of Fe (10,000 mg/kg) in shoot tissues (Doku et al., 2024; Kahangwa et al., 2021; Kang et al., 2018; Petelka et al., 2019). The aggressive uptake of Fe is linked to the sequestrating activities of plant growth-promoting rhizobacteria and siderophores, which serve as a mechanism for overcoming heavy metal-induced stress (Bomfim et al., 2021; Liu et al., 2024; Yan et al., 2020). Moreover, they have been known to secrete siderophores, phenolics, and organic acids that target the sequestration of divalent ions (Cu^2+^, Zn^2+^, Mn^2+^ and Ca^2+^), chiefly Fe, to maintain vital metabolic processes (Lee et al., 2023; Narayanan and Ma, 2023; Qu et al., 2020; Yang et al., 2022).

The high rhizospheric concentrations of Mn (<3,000 mg/kg) in pure tailing and 1:1 treatments, coupled with an acidic rhizosphere, favoured preferential initial accumulation in root tissues despite the competitive limitation imposed by elevated Fe levels (Abubakari et al., 2022; Garcia et al., 2020; Dey et al., 2023; Yu et al., 2019; Yang et al., 2008). Despite the high mobility of As and Cd in plant tissues, they showed preferential retention in the root, which conflicts with other reports (Kahangwa et al., 2021; Muehe et al., 2015; Saldarriaga et al., 2023; Yan et al., 2022). The minimal background concentrations, vacuolar compartmentalisation in root tissues, and mechanisms which limit translocation to protect other organs and photosynthetic tissues could account for poor shoot accumulation of Cd and As (Liu et al., 2023; Luo and Zhang, 2021; Geng et al., 2023; Khan et al., 2021; Nabi et al., 2021; Schneider et al., 2017). Metal accumulation generally increased over time (H0 < H1 < H2 < H3), indicating a progressive uptake capacity as plants matured and root systems expanded.

### 4.3 Bacterial community assembly under metal selection pressure

The results show changing dynamics in the bacterial abundance and diversity; an initial decrease (H0–H1) followed by an increase (H1–H2) and a decrease (H2–H3) demonstrates a deterministic community assembly of bacterial communities in the rhizosphere of *L. leucocephala* under metal stress and oligotrophic conditions during phytoremediation (Khalid et al., 2023; Muratova et al., 2023; Park et al., 2023; Sun et al., 2024). Specifically, the initial community disruption (H0–H1) was followed by selective enrichment of metal-tolerant taxa (*Nocardioides* and *Streptomyces*), representing a notable ecological response to extreme environmental filtering. It is worth noting that the overall bacterial community abundance and diversity are lower compared to a previous report of tailings and rhizosphere of *L. leucocephala*, limiting the possibilities of remediating tropical gold tailings (Gagnon et al., 2020; Doku et al., 2024; Qian et al., 2023; Trovão et al., 2024). The collective adverse impacts of elevated metal levels, acidification, and depleted nutrient levels, as shown by the correlation heatmap, favour deterministic assembly processes that sustain microbial survival and metal sequestration over time (Lei et al., 2024; Maretto et al., 2022; Romero et al., 2021; Sun et al., 2022). Again, the recovery of bacterial diversity at the later stages of experimentation (H2–H3) in tailings was concomitant with the depletion of metal ions in the rhizosphere and subsequent uptake in root tissues, thus reflecting the successful adaptation and niche differentiation among surviving taxa. More so, the temporal succession pattern suggests that established phytoremediation systems may achieve greater microbial stability and functional capacity over time. Beta diversity analysis revealed significant differences among treatments, suggesting that contamination intensity drives shifts in bacterial community assembly (Guo et al., 2019; Huang et al., 2021; Saldarriaga et al., 2023; Yu et al., 2022).

### 4.4 Functional roles of dominant bacterial taxa

#### 4.4.1 Key functional bacterial phyla during phytoremediation

The dominance of *Actinobacteria*, *Proteobacteria* and *Acidobacteria* in the rhizosphere is consistent with other reports that studied microbial dynamics during phytoremediation of metal-contaminated media (Gao et al., 2021; Luo et al., 2022; Muratova et al., 2023; Yang et al., 2022). Specifically, *Actinobacteria* demonstrated consistent dominance, with an approximate relative abundance of 35% across all treatments, which is attributed to a myriad of factors that sustain exceptional stress tolerance and contribute to plant growth (Gao et al., 2021; Guo et al., 2019; Liu et al., 2024). More so, predicted functions such as secretion of siderophores, auxins production, biofilm formation and colonisation were associated with predominant *Actinobacteria* in metalliferous settings (pure tailings and 1:1), which concurs with their known capabilities under extreme conditions (Alvarez et al., 2017; Behera and Das, 2023; Qian et al., 2023).

#### 4.4.2 Key functional bacterial genera during phytoremediation

The relative abundance of prominent bacterial genera, *including Gaiella, Arthrobacter, Nocardioides, and Streptomyces,* in the rhizosphere showed unique patterns in response to metal contamination and temporal gradients (Alvarez et al., 2017; Hanbo et al., 2004; Nkongolo et al., 2025; Nosalova et al., 2022). As the most critical genus based on abundance and network centrality analysis, the populations of *Arthrobacter* grew along the temporal gradient and with increasing contamination. In line with predicted functions, this could indicate selective recruitment to enhance metal sorption, accumulation and transformation, which is a constitutive trait of hyperaccumulating plants (Park et al., 2023; Visioli et al., 2015; Wu et al., 2020). The initial proliferation of *Nocardioides* and *Streptomyces* could represent an early colonisation adaptive mechanism to mitigate environmental stress, based on concomitant predicted functions such as secretion of plant-growth hormones, metal scavengers, and nitrogen fixation (Ali et al., 2021; Li et al., 2025; Xin et al., 2023). The notable continual proliferation of *Nocardioides* at the second harvest suggests the ability of the rhizosphere microbiome to moderate its composition to improve nutrient levels in oligotrophic environments, tolerate metal-induced toxicity, and facilitate sequestration (Geng et al., 2023; Li et al., 2025; Wang et al., 2022). Unlike *Arthrobacter*, *Gaiella* populations generally decreased across all treatments; however, these bacteria showed strong associations with metal (Fe, Mn, Cd, and As) sequestration into plant tissues. The spike in *Arthrobacter* populations at the final harvest in pure tailings concomitant with the accumulation of metals in the shoot could be attributed to its transforming into non-toxic forms, thus inducing metal uptake and translocation into plant tissues (Asatiani et al., 2018; Guo D, et al., 2019; Khoshru et al., 2023; Román-Ponce et al., 2018; Saharan et al., 2023).

#### 4.4.3 Ecological network implications for system stability

The identification of keystone species through network analysis provides crucial insights for the manipulation strategies. The topological features unveil the relevance of low-abundance minor genera such as *Skermanella* and *Chelatococcus* in maintaining key ecological functions attributed to their capacity to improve nitrogen levels and facilitate metal sequestration despite their sensitivity to metal contamination in oligotrophic environments (Li et al., 2016; Li et al., 2024; Paul et al., 2024; Xu et al., 2024). Thus, *Arthrobacter*, *Skermanella* and *Chelatococcus* represent high-priority targets for developing bacterial inoculants, as their network positions suggest a disproportionate influence on community stability and function (Bhat et al., 2022; Fernández-González et al., 2017; Galani et al., 2024). Moreover, the within-module roles of *Actinobacteria*, *Proteobacteria*, and *Acidobacteria* as major connectors and module hubs indicate their specialised community functions, promoting nutrient cycling, metal transformation, and plant support (Che et al., 2022; Duan et al., 2021; Sun et al., 2024). The peripheral network roles of *Chloroflexi*, *Firmicutes*, *Gemmatimonadetes*, and *Bacteroidetes* distinguish their activities in maintaining network integrity and functions in the remediation of mine tailings (Deng et al., 2012; Guo et al., 2022; Meyer et al., 2020).

### 4.5 Practical implications for mine tailing rehabilitation

The findings of this research are relevant to improving microbiome-enhanced phytoremediation strategies, including the development of bacterial inoculants using *Arthrobacter*, *Gaiella*, *Streptomyces*, and *Nocardioides*. Additionally, improving soil nutrient levels and pH promotes the selective growth of beneficial microbiomes and the utilisation of microbial dynamics to monitor the progress of phytoremediation efforts.

### 4.6 Study limitations and future perspectives

While the study provides valuable insights into bacterial community diversity during the phytoremediation of tailings, initial nursing in the greenhouse may not accurately capture field conditions, such as weather variability and soil heterogeneity. Moreover, using composite samples for metagemoic profiling limit the robustness of statistical analysis of bacterial communities, whereas predicted functional roles may not accurately capture the exact contributions provided by culture-based methods. Again, the three-month sampling period intervals targeted long-term dynamics but may have missed shorter-term dynamics important for understanding community assembly.

## 5 Conclusion

This study provides comprehensive insights into the rhizosphere dynamics that support the phytoremediation of multiple metal-contaminated mine tailings by *Leucaena leucocephala*. Thus, our findings demonstrate that successful phytoremediation depends on coordinated plant-microbe adaptations, such as acidification and nutrient cycling, that alter rhizosphere chemistry, selectively enrich metal-tolerant bacterial taxa, and improve metal uptake into plant tissues.

## Data Availability

The raw data supporting the conclusions of this article will be made available by the authors, without undue reservation.
