# Supplementary material for: Rhizosphere microbiome assembly drives metal sequestration in Leucaena leucocephala during tailing phytoremediation
Source: Front Microbiol. 2026 Feb 13;17:1745018. doi: 10.3389/fmicb.2026.1745018 (PMC12946116; doi:10.3389/fmicb.2026.1745018)
Supplement: Supplementary file 1 [file Data_Sheet_1.docx]

**Supplementary Materials**

**Figures**


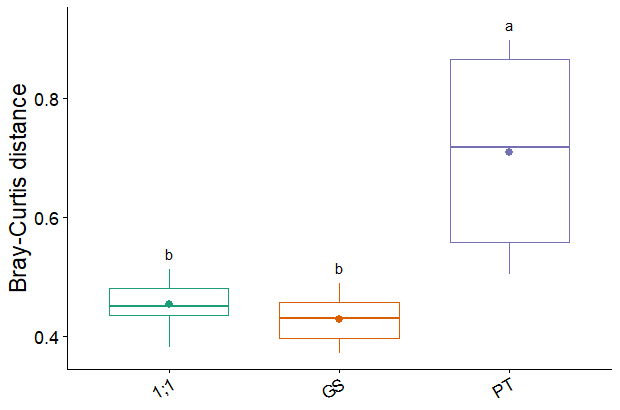


Supplementary Figure 1. Boxplot of beta diversity of bacterial communities in treatments showing significant differences (p < 0.05; PERMANOVA)


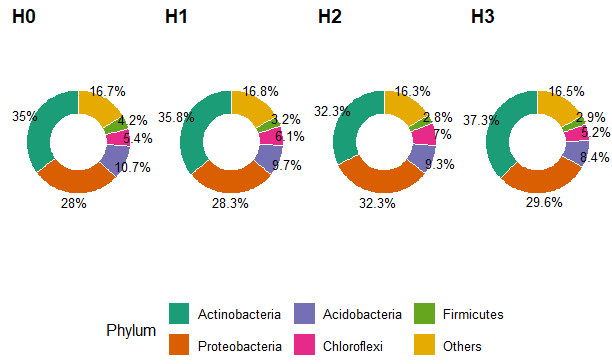


Supplementary Figure 2a. Doughnut plot of bacterial phyla in the rhizosphere during phytoremediation


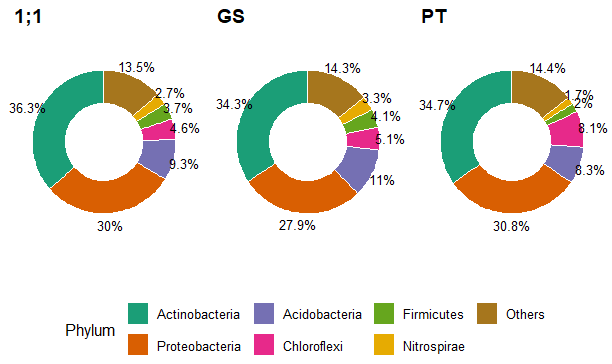


Supplementary Figure 2b. Doughnut plot of bacterial phyla in the rhizosphere during phytoremediation


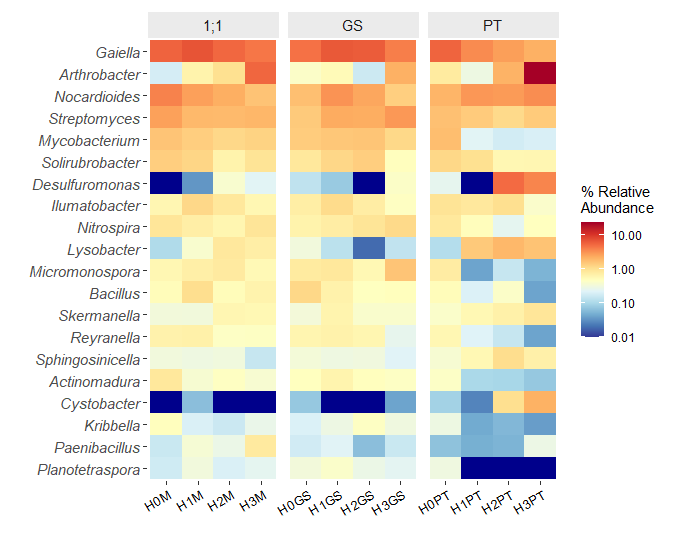


Figure 3. Heatmap of the relative abundance of bacterial genera in the rhizosphere during phytoremediation.


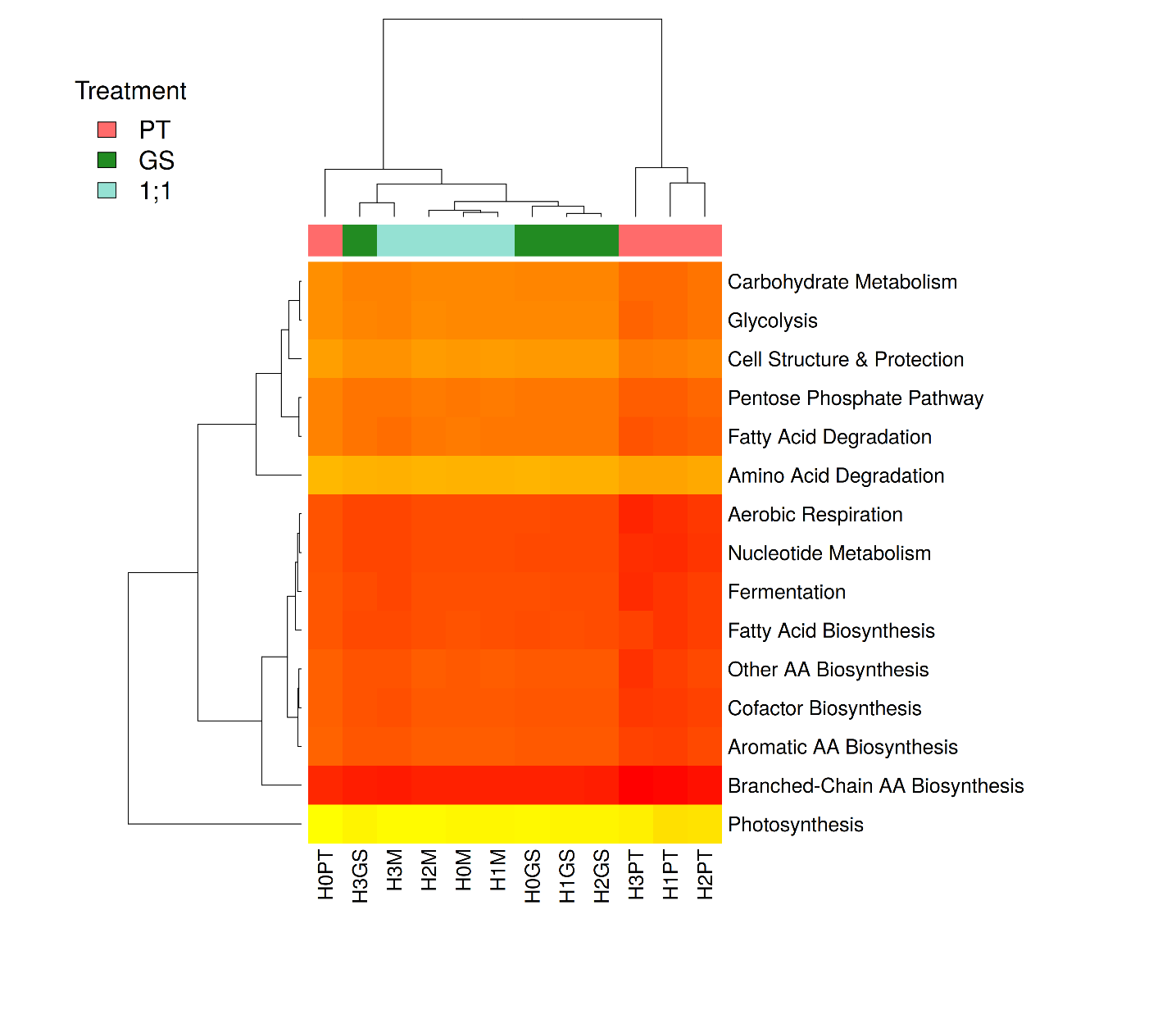


Supplementary Figure 4. Heatmap of major predicted functions in treatments


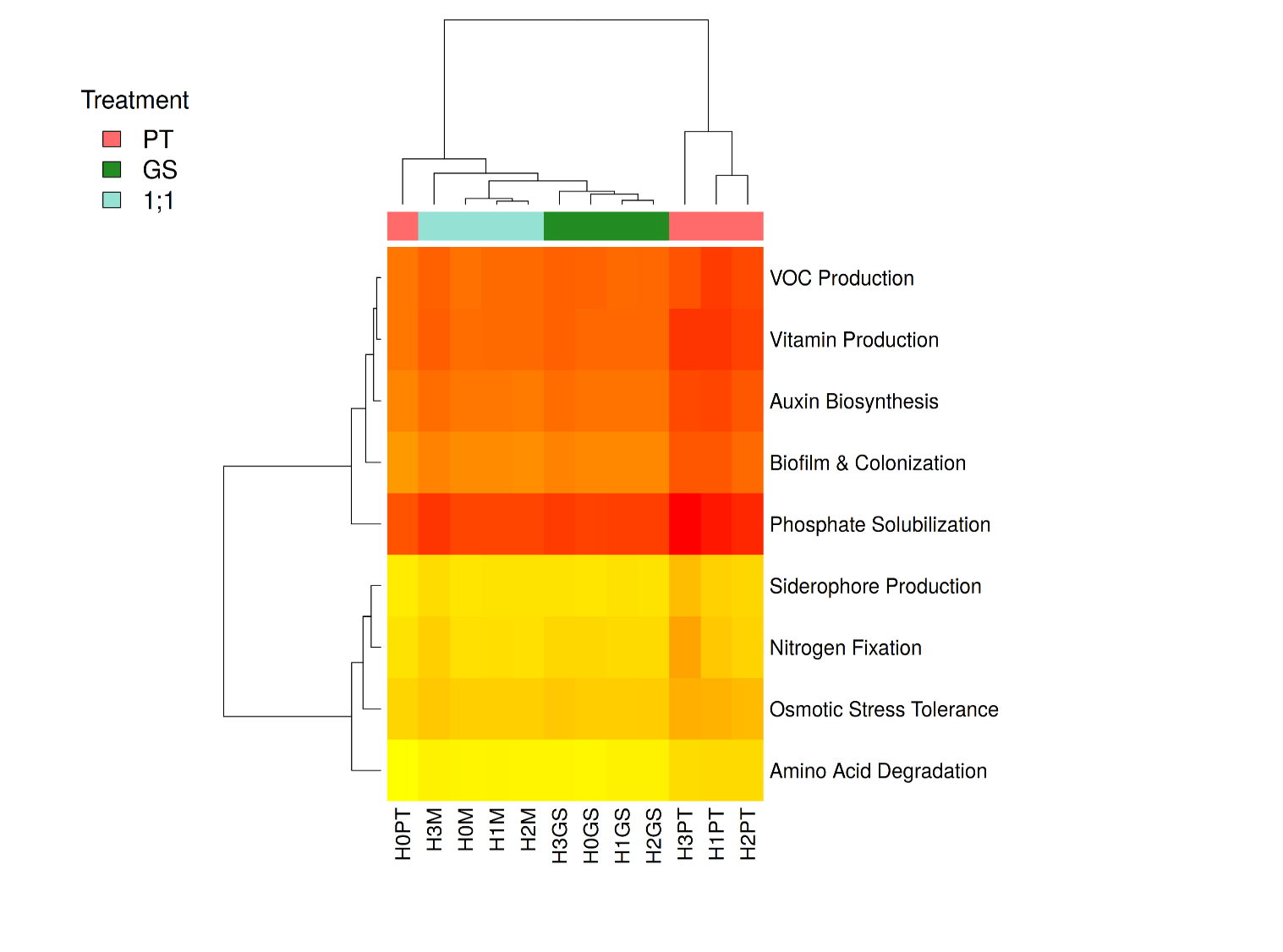


Supplementary Figure 5. Heatmap of plant growth-promoting predicted functions in treatments


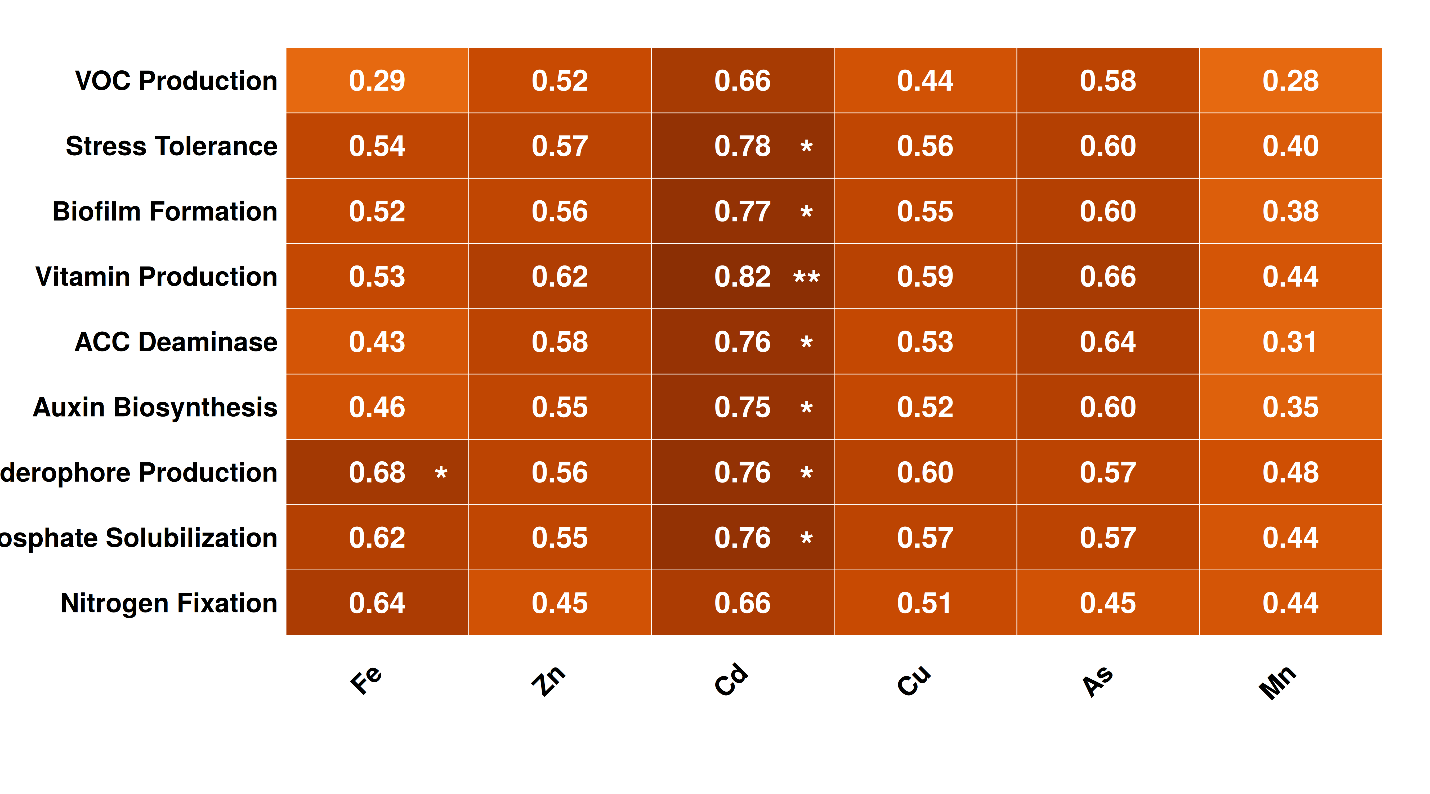
Supplementary Figure 6. Correlation heatmap of predicted functions and shoot accumulation


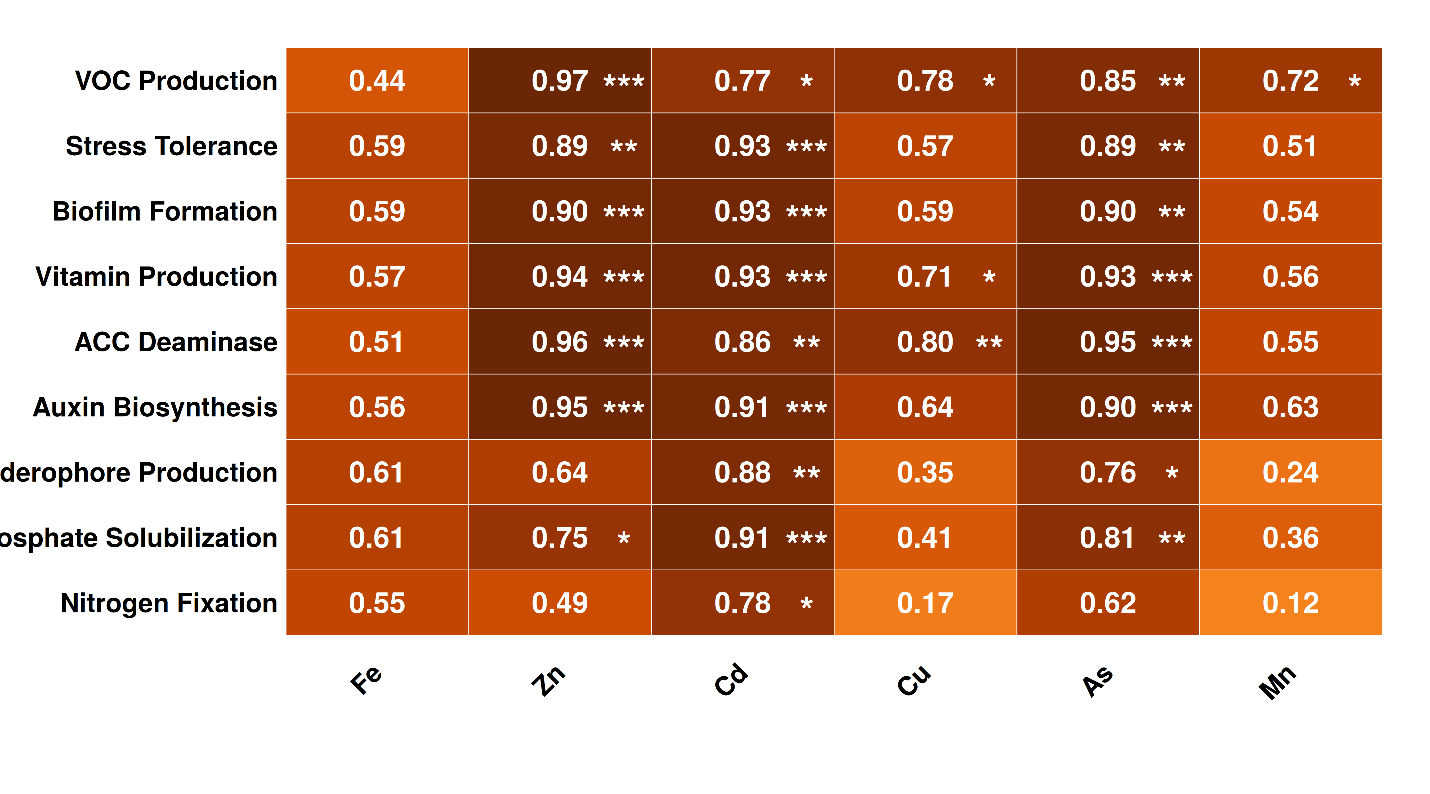
Supplementary Figure 7. Correlation heatmap of predicted functions and root accumulation


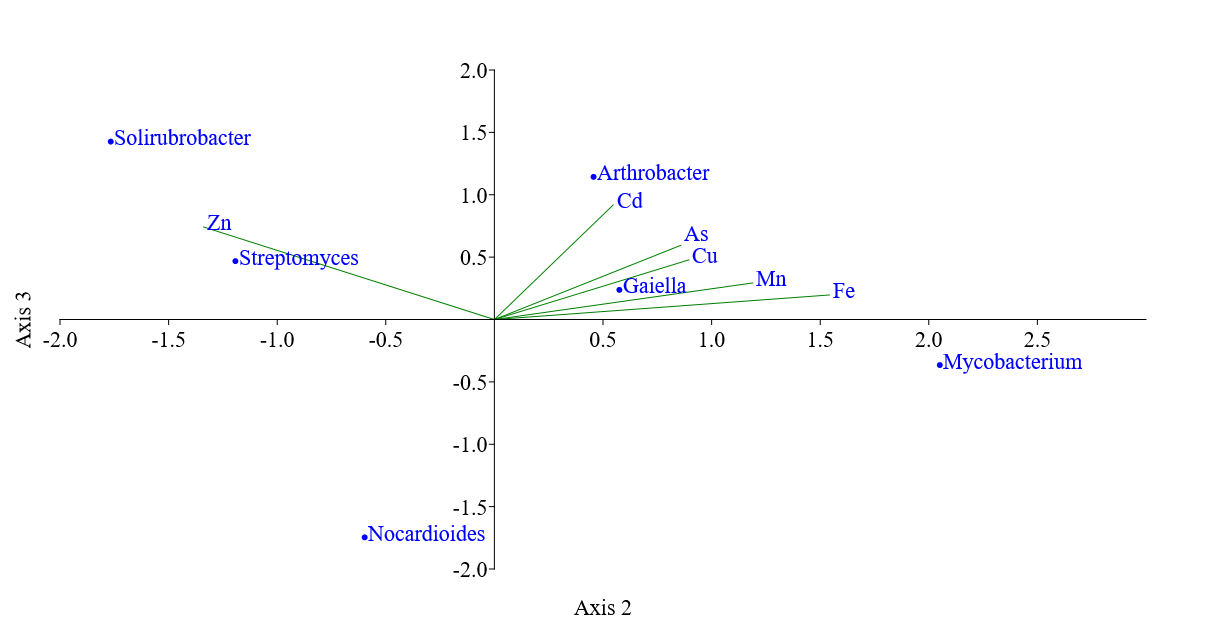


Supplementary Figure 8. Canonical correspondence analysis showing bacterial genera associated with metal uptake in the root


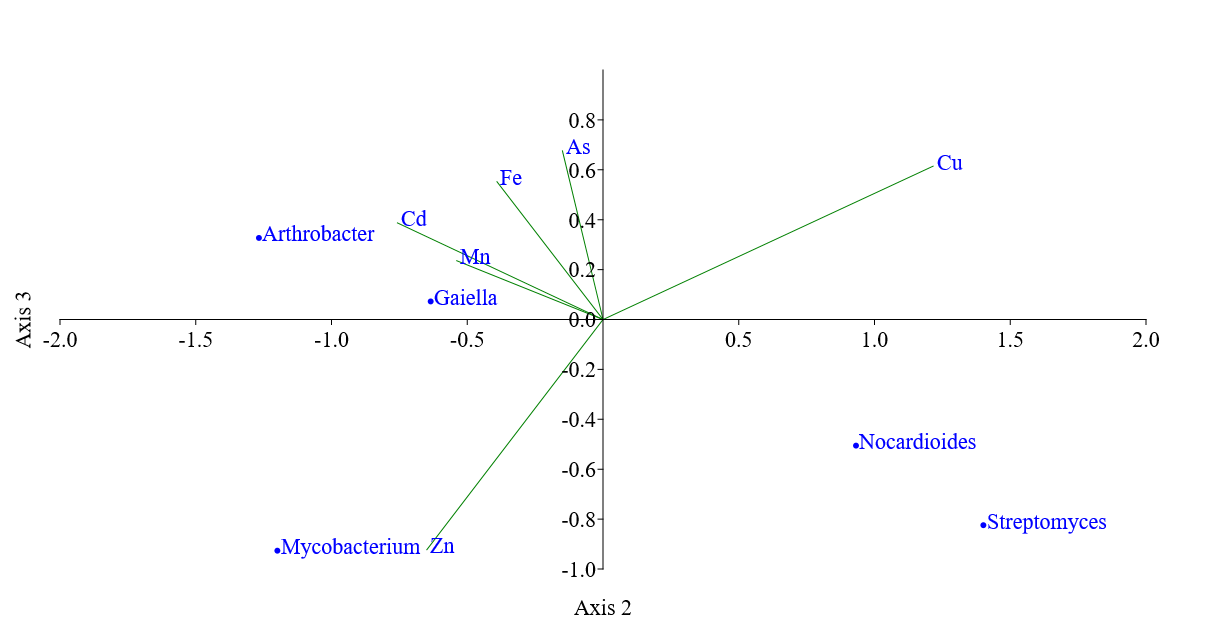


Supplementary Figure 9. Canonical correspondence analysis showing bacterial genera associated with metal uptake in the shoot


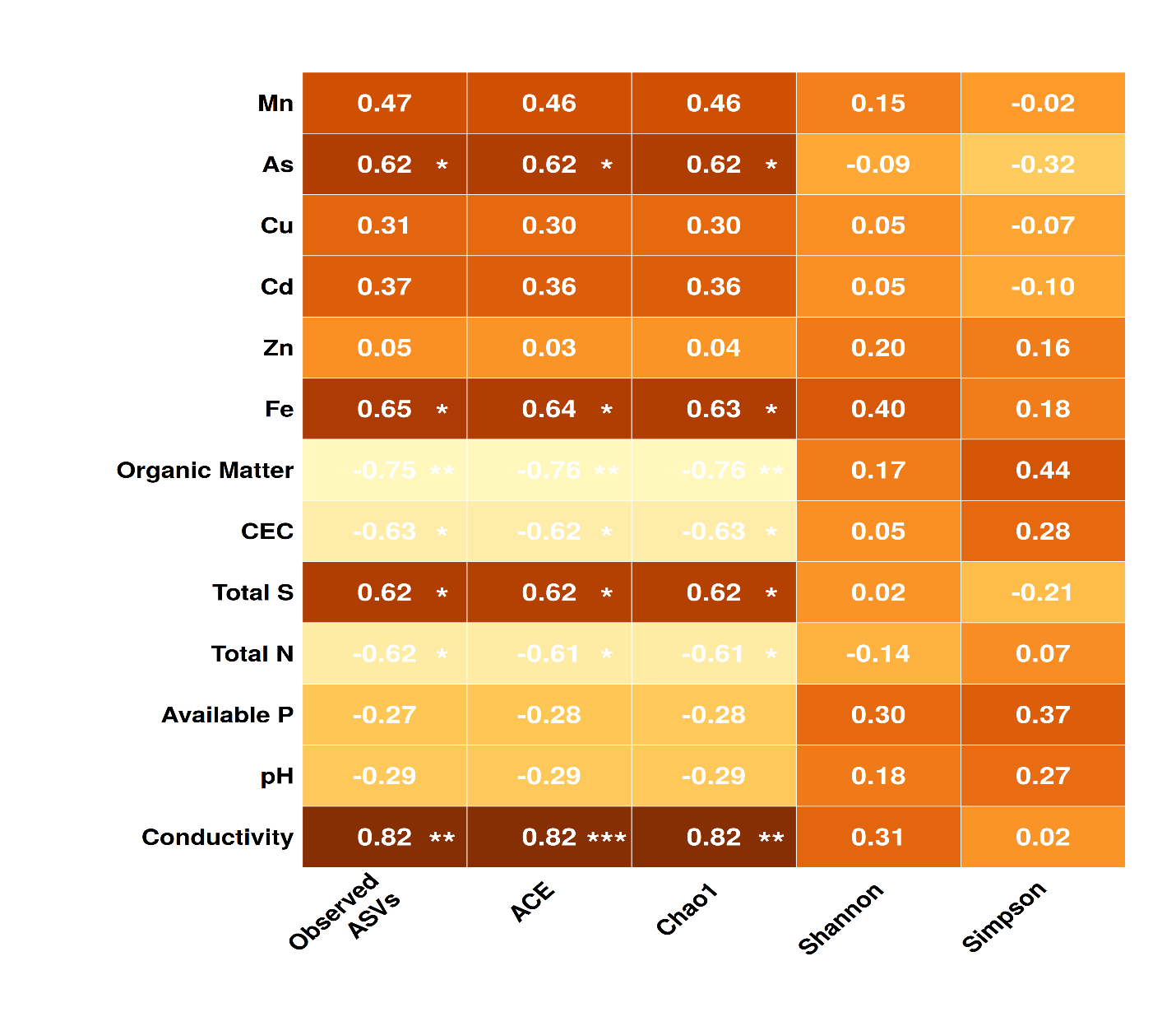


Supplementary Figure 10. Correlation heatmap of environmental factors and bacterial diversity


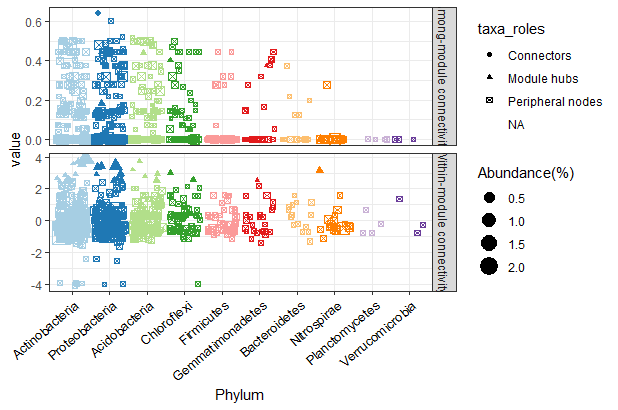


Supplementary Figure 11. Network roles of predominant phyla in the rhizosphere during phytoremediation
