# Supplementary material for: Rhizosphere microbiome assembly drives metal sequestration in Leucaena leucocephala during tailing phytoremediation
Source: Front Microbiol. 2026 Feb 13;17:1745018. doi: 10.3389/fmicb.2026.1745018 (PMC12946116; doi:10.3389/fmicb.2026.1745018)
Supplement: Supplementary file 2 [file Data_Sheet_2.docx]

Supplementary Table 1. Mean heavy metal concentrations in the root of *L. Leucocephala* during phytoremediation

| **Treatment** | **Harvest** | **Fe** | **Zn** | **Cd** | **Cu** | **As** | **Mn** |
| --- | --- | --- | --- | --- | --- | --- | --- |
| Garden soil | H1 | 246.70±0.42c | 16.90±0.14b | 0.010±0.001a | 19.92±0.11a | 0.51±0.01b | 45.35±0.21b |
|  | H2 | 25.70±0.14a | 22.30±0.14c | 0.076±0.007b | 28.50±0.71b | 0.41±0.01a | 43.90±1.14b |
|  | H3 | 156.70±0.42b | 13.35±0.21a | 0.015±0.004a | 35.88±0.17c | 0.41±0.01a | 40.90±0.40a |
| 1:1 | H1 | 7038.70±0.43f | 198.65±0.50f | 0.93±0.02e | 174.09±0.14e | 45.89±0.16e | 5664.50±1.75g |
|  | H2 | 9215.35±0.21g | 122.90±0.15e | 0.60±0.08c | 121.94±0.08d | 22.25±0.08c | 555.30±1.41c |
|  | H3 | 4595.80±0.29e | 112.90±0.50d | 1.26±0.05f | 243.43±0.10g | 34.00±0.01d | 1465.10±1.40d |
| Pure tailings | H1 | 9736.90±0.14h | 2929.80±0.28i | 2.89±0.02h | 383.88±0.17h | 123.38±0.17f | 12279.20±1.13h |
|  | H2 | 4476.25±0.35d | 1937.80±0.30h | 1.85±0.14g | 593.35±0.21i | 125.91±0.15g | 2083.35±1.21e |
|  | H3 | 11032.90±0.14i | 1668.90±0.15g | 3.34±0.02i | 206.25±0.35f | 125.39±0.15g | 2168.35±1.30f |

Mean values (means ± standard error) with different letters indicate significant differences (p < 0.05, Tukey's HSD test).

Supplementary Table 2. Mean heavy metal concentrations in the shoot of *L. Leucocephala* during phytoremediation

| **Treatment** | **Harvest** | **Fe** | **Zn** | **Cd** | **Cu** | **As** | **Mn** |
| --- | --- | --- | --- | --- | --- | --- | --- |
| Garden soil | H1 | 366.30±0.42b | 24.35±0.21a | 0.015±0.002a | 29.26±0.37a | 0.75±0.01b | 6.79±0.05ab |
|  | H2 | 380.25±0.21c | 38.25±0.35b | 0.015±0.003a | 42.25±0.35b | 0.62±0.01a | 6.53±0.23a |
|  | H3 | 228.40±0.28a | 198.90±1.40c | 0.021±0.002b | 53.38±0.03c | 0.61±0.01a | 6.05±0.16a |
| 1:1 | H1 | 6893.20±2.83e | 2906.45±1.07d | 0.56±0.02c | 261.06±0.08d | 27.53±0.03c | 832.90±1.40c |
|  | H2 | 9484.65±2.12g | 3859.60±1.56e | 0.58±0.01d | 275.65±0.35e | 31.49±0.01c | 2496.25±1.35f |
|  | H3 | 10557.80±2.80h | 4394.70±1.42h | 1.40±0.02g | 309.25±1.30i | 33.29±0.01e | 4558.90±1.14h |
| Pure tailings | H1 | 6714.50±1.70d | 4016.70±1.40f | 1.32±0.01f | 275.82±1.25f | 37.96±0.05f | 1917.30±1.40d |
|  | H2 | 7592.70±1.42f | 4393.10±1.42g | 1.51±0.02h | 289.90±1.41g | 38.81±0.12g | 2109.80±1.28e |
|  | H3 | 14605.10±1.40i | 4699.10±1.40i | 1.79±0.02i | 365.02±1.35h | 39.75±0.35h | 3124.90±1.14g |

Mean values (means ± standard error) with different letters indicate significant differences (p < 0.05, Tukey's HSD test).

Supplementary Table 3. Alpha diversity of bacterial communities in the rhizosphere during phytoremediation

| **Treatment** | **Harvest** | **Alpha diversity indices of ASVs** | | | | |
| --- | --- | --- | --- | --- | --- | --- |
|  |  | **Observed** | **ACE** | **Chao1** | **Shannon** | **Simpson** |
| **Garden soil** | H0 | 1284 | 1291.84 | 1295.69 | 6.69 | 0.998 |
|  | H1 | 1275 | 1282.86 | 1282.86 | 6.71 | 0.998 |
|  | H2 | 1109 | 1111.59 | 1111.29 | 6.56 | 0.998 |
|  | H3 | 1192 | 1198.88 | 1205.18 | 6.55 | 0.997 |
| **1:1** | H0 | 1258 | 1253.99 | 1258.00 | 6.66 | 0.998 |
|  | H1 | 1242 | 1248.73 | 1251.02 | 6.65 | 0.998 |
|  | H2 | 1266 | 1275.81 | 1277.92 | 6.67 | 0.998 |
|  | H3 | 1268 | 1278.97 | 1290.02 | 6.49 | 0.995 |
| **Pure tailings** | H0 | 1294 | 1301.48 | 1307.05 | 6.69 | 0.998 |
|  | H1 | 1952 | 1996.76 | 2017.61 | 6.91 | 0.996 |
|  | H2 | 1623 | 1643.30 | 1651.02 | 6.77 | 0.997 |
|  | H3 | 1354 | 1379.73 | 1391.81 | 5.63 | 0.960 |

Supplementary Table 4. Overall network characteristics of bacterial communities

| Total nodes | 81 |
| --- | --- |
| Total links | 678 |
| Maximal degree | 40 |
| **Nodes with max degree** | **OTU32** |
| Centralisation of degree (CD) | 0.298 |
| Maximal betweenness | 210.450 |
| **Nodes with max betweenness** | **OTU179** |
| Centralisation of betweenness (CB) | 0.055 |
| Maximal stress centrality | 1433 |
| Nodes with max stress centrality | OTU179 |
| Centralisation of stress centrality (CS) | 0.346 |
| Maximal eigenvector centrality | 0.235 |
| **Nodes with max eigenvector centrality** | **OTU32** |
| Centralisation of eigenvector centrality (CE) | 0.148 |
| Density (D) | 0.209 |
| Reciprocity | 1 |
| Transitivity (Trans) | 0.444 |
| Connectedness (Con) | 1 |
| Efficiency | 0.801 |
| Hierarchy | 0 |
| Lubness | 1 |

Supplementary Table 5a. Contribution of bacterial genera in the first modular network

| Genus | Node degree | Node betweenness | Node stress | Eigenvector centrality | | Clustering coefficient |
| --- | --- | --- | --- | --- | --- | --- |
| *Arthrobacter* | 40 | 90.74 | 987 | 0.24 | 0.47 | |
| *Sorangium* | 35 | 41.58 | 632 | 0.22 | 0.55 | |
| *Haliangium* | 35 | 61.27 | 753 | 0.22 | 0.54 | |
| *Burkholderia* | 34 | 67.95 | 722 | 0.21 | 0.54 | |
| *Pseudomonas* | 30 | 43.60 | 531 | 0.19 | 0.59 | |
| *Haliea* | 36 | 147.63 | 1098 | 0.19 | 0.33 | |
| *Lacibacter* | 30 | 66.29 | 725 | 0.18 | 0.44 | |
| *Pasteuria* | 27 | 34.78 | 338 | 0.18 | 0.62 | |
| *Actinomycetospora* | 26 | 12.93 | 281 | 0.18 | 0.66 | |
| *Nitrosospira* | 28 | 44.16 | 623 | 0.18 | 0.52 | |
| *Aneurinibacillus* | 30 | 58.62 | 696 | 0.18 | 0.42 | |
| *Trachelomonas* | 28 | 50.30 | 685 | 0.18 | 0.49 | |
| *Ilumatobacter* | 27 | 25.75 | 464 | 0.17 | 0.52 | |
| *Unkown* | 30 | 110.86 | 883 | 0.17 | 0.47 | |
| *Thermomonas* | 26 | 21.87 | 426 | 0.17 | 0.51 | |
| *Solitalea* | 28 | 56.11 | 535 | 0.16 | 0.40 | |
| *Elizabethkingia* | 29 | 129.42 | 1183 | 0.16 | 0.37 | |

Supplementary Table 5b. Contribution of bacterial genera in the second modular network

| Genus | Node degree | Node betweenness | Node stress | Eigenvector centrality | Clustering Coefficient |
| --- | --- | --- | --- | --- | --- |
| *Chelatococcus* | 29 | 98.26 | 769 | 0.16 | 0.43 |
| *Pirellula* | 27 | 139.61 | 1063 | 0.14 | 0.30 |
| *Pedobacter* | 20 | 75.93 | 585 | 0.08 | 0.21 |
| *Laceyella* | 15 | 20.04 | 205 | 0.08 | 0.42 |
| *Kineosporia* | 13 | 43.19 | 332 | 0.06 | 0.37 |
| *Seinonella* | 13 | 38.61 | 266 | 0.05 | 0.28 |
| *Clostridium* | 13 | 44.37 | 323 | 0.05 | 0.23 |
| *Altererythrobacter* | 17 | 48.98 | 378 | 0.04 | 0.36 |
| *Georgfuchsia* | 8 | 18.46 | 130 | 0.04 | 0.32 |
| *Haliscomenobacter* | 15 | 47.31 | 356 | 0.04 | 0.38 |
| *Salinarimonas* | 12 | 18.86 | 164 | 0.04 | 0.38 |
| *Fimbriimonas* | 7 | 18.77 | 135 | 0.03 | 0.24 |
| *Haloferula* | 13 | 51.13 | 317 | 0.03 | 0.35 |
| *Actinomadura* | 6 | 7.12 | 55 | 0.02 | 0.47 |
| *Acinetobacter* | 7 | 16.73 | 98 | 0.02 | 0.43 |
| *Belnapia* | 7 | 3.35 | 38 | 0.02 | 0.62 |
| *Plesiocystis* | 4 | 3.19 | 18 | 0.01 | 0.33 |
| *Symbiobacterium* | 2 | 0.14 | 1 | 0.01 | 0.00 |
| *Niastella* | 5 | 5.17 | 28 | 0.01 | 0.50 |

Supplementary Table 5c. Contribution of bacterial genera in the third modular network

| Genus | Node degree | Node betweenness | Node stress | Eigenvector centrality | Clustering coefficient |
| --- | --- | --- | --- | --- | --- |
| *Skermanella* | 37 | 210.45 | 1433 | 0.18 | 0.30 |
| *Massilia* | 21 | 67.39 | 519 | 0.11 | 0.44 |
| *Thiocystis* | 21 | 55.78 | 491 | 0.09 | 0.40 |
| *Alkanindiges* | 19 | 85.94 | 731 | 0.09 | 0.25 |
| *Entotheonella* | 15 | 21.70 | 247 | 0.08 | 0.50 |
| *Spongiibacter* | 12 | 24.17 | 225 | 0.05 | 0.33 |
| *Inquilinus* | 10 | 9.21 | 74 | 0.05 | 0.40 |
| *Desulfopila* | 13 | 17.83 | 154 | 0.04 | 0.44 |
| *Antarcticicola* | 14 | 49.57 | 328 | 0.04 | 0.22 |
| *Flavihumibacter* | 7 | 7.79 | 66 | 0.04 | 0.33 |
| *Thermoflavimicrobium* | 9 | 6.89 | 67 | 0.03 | 0.53 |
| *Azoarcus* | 9 | 23.35 | 123 | 0.03 | 0.22 |
| *Caulobacter* | 6 | 1.08 | 9 | 0.03 | 0.73 |
| *Alloactinosynnema* | 8 | 19.09 | 125 | 0.03 | 0.29 |
| *Bacillus* | 9 | 23.50 | 165 | 0.03 | 0.31 |
| *Caldimonas* | 5 | 2.24 | 21 | 0.02 | 0.30 |
| *Filomicrobium* | 3 | 2.89 | 13 | 0.00 | 0.00 |

Supplementary Table 6a. Bioaccumulation Factor of Heavy metals in the root

| **Treatment** | **Harvest** | **Fe** | **Zn** | **Cd** | **Cu** | **As** | **Mn** |
| --- | --- | --- | --- | --- | --- | --- | --- |
| GS | H1 | 1.14 | 2.50 | 0.04 | 0.51 | 0.20 | 0.12 |
|  | H2 | 1.40 | 6.59 | 0.03 | 0.86 | 0.17 | 0.18 |
|  | H3 | 1.01 | 5.59 | 0.04 | 1.68 | 0.17 | 0.19 |
| 1:1 | H1 | 21.47 | 298.10 | 1.44 | 4.51 | 7.43 | 14.12 |
|  | H2 | 35.00 | 665.45 | 1.10 | 5.59 | 8.50 | 70.32 |
|  | H3 | 46.72 | 123.62 | 2.56 | 11.51 | 9.08 | 140.27 |
| PT | H1 | 20.92 | 411.97 | 3.37 | 4.77 | 10.25 | 32.50 |
|  | H2 | 28.02 | 757.43 | 2.85 | 5.87 | 10.48 | 59.43 |
|  | H3 | 64.62 | 132.18 | 3.28 | 9.76 | 10.85 | 96.15 |

Supplementary Table 6b. Bioaccumulation Factor of Heavy metals in the shoot

| **Treatment** | **Harvest** | **Fe** | **Zn** | **Cd** | **Cu** | **As** | **Mn** |
| --- | --- | --- | --- | --- | --- | --- | --- |
| GS | H1 | 0.77 | 1.73 | 0.02 | 0.34 | 0.14 | 0.77 |
|  | H2 | 0.09 | 3.84 | 0.14 | 0.58 | 0.11 | 1.24 |
|  | H3 | 0.69 | 0.38 | 0.03 | 1.13 | 0.11 | 1.26 |
| 1:1 | H1 | 21.93 | 300.49 | 2.38 | 3.01 | 12.39 | 96.01 |
|  | H2 | 34.00 | 334.10 | 1.12 | 2.47 | 6.00 | 15.64 |
|  | H3 | 20.34 | 46.95 | 2.31 | 7.68 | 9.28 | 45.08 |
| PT | H1 | 30.33 | 20.37 | 7.42 | 6.64 | 33.30 | 208.12 |
|  | H2 | 16.52 | 21.19 | 3.49 | 12.02 | 33.98 | 58.69 |
|  | H3 | 48.82 | 3.18 | 6.12 | 6.51 | 34.21 | 66.72 |

Supplementary Table 7. Translocation factor of heavy metals

| **Treatment** | **Harvest** | **Fe** | **Zn** | **Cd** | **Cu** | **As** | **Mn** |
| --- | --- | --- | --- | --- | --- | --- | --- |
| GS | H1 | 1.48 | 1.44 | 1.54 | 1.47 | 1.49 | 0.15 |
|  | H2 | 14.80 | 1.72 | 1.52 | 1.48 | 1.51 | 0.15 |
|  | H3 | 1.46 | 14.90 | 1.41 | 1.49 | 1.49 | 0.15 |
| 1:1 | H1 | 0.98 | 0.99 | 0.60 | 1.50 | 0.60 | 0.15 |
|  | H2 | 0.97 | 0.50 | 1.02 | 0.44 | 0.71 | 0.22 |
|  | H3 | 2.30 | 2.63 | 1.11 | 1.50 | 0.98 | 3.11 |
| PT | H1 | 0.69 | 20.22 | 0.45 | 0.72 | 0.31 | 0.16 |
|  | H2 | 1.70 | 35.75 | 0.82 | 0.49 | 0.31 | 1.01 |
|  | H3 | 1.32 | 41.62 | 0.54 | 1.50 | 0.32 | 1.44 |
